# Brewer’s Spent Grain as a Source of Proteins and Valuable Polysaccharides

**DOI:** 10.3390/foods15101701

**Published:** 2026-05-12

**Authors:** Andrea Están, Susana Simal, Valeria Eim, Juan Cárcel, Adda Ibañez, Mónica Umaña

**Affiliations:** 1 Department of Chemistry, University of the Balearic Islands, Ctra. Valldemossa, km 7.5, 07122 Palma, Spain; andrea.estan@uib.es (A.E.); susana.simal@uib.es (S.S.); valeria.eim@uib.es (V.E.); 2 Analysis and Simulation of Agro-Food Processes Group, Food Engineering Research Institute-FoodUPV, Universitat Politècnica de València, 46022 Valencia, Spain; jcarcel@tal.upv.es (J.C.); aibadau@upvnet.upv.es (A.I.)

**Keywords:** brewery waste, protein, ultrasound, polysaccharides, hemicellulose, mathematical modeling

## Abstract

Brewer’s spent grain (BSG) is an abundant by-product rich in proteins and polysaccharides. This study evaluated ultrasound (US) to enhance alkaline protein extraction in 110 mM NaOH and to obtain a polysaccharide-enriched residue, with mechanical agitation (AG) as the control. First, 40 min extraction curves were evaluated at 25 and 50 °C and fitted to the Weibull model. At 25 °C, US increased the 40 min protein yield (40.8 ± 0.1 g/100 g initial protein) by 2.5-fold compared with AG; heating increased AG yield, whereas US showed negligible temperature sensitivity between 25 and 50 °C. Subsequently, extractions were performed at 25 °C in one or two 20 min cycles using fresh solvent. Extracts were recovered by pH-shift precipitation and freeze-dried. US maximised protein recovery (47.6 ± 0.1 g/100 g initial protein after two cycles) but co-extracted other compounds, reducing purity. AG extracts showed higher emulsifying activity (up to 9.6 ± 0.1 m^2^·g^−1^), while US increased emulsion stability (up to 46 min). US residues showed up to ~35% lower glucose content than BSG (42 ± 2 g/100 g dry matter for BSG) and higher arabinoxylans (up to 23.5 ± 0.6 g/100 g dry matter; ~2.4-fold BSG), supporting a dual valorisation route based on the production of a protein-rich extract and an arabinoxylans-rich concentrate.

## 1. Introduction

Brewer’s spent grain (BSG) is the main solid by-product of the brewing industry. It is generated during lautering after mashing, when the wort is separated from the insoluble fraction of malted barley. BSG is produced at approximately 20 kg per hL of beer, resulting in an estimated global production of approximately 40 Mt per year [1,2]. Despite its high availability, BSG is primarily used for low-value applications, such as animal feed and energy, and remains underutilised as a food-grade ingredient [3].

At the same time, BSG is attracting increasing attention as a sustainable source of plant-based proteins, in line with the global rise in protein demand and the need to transition to more resource-efficient food systems [4]. Upcycling agro-industrial residues into food ingredients can reduce waste generation, support circular economy models, and contribute to the diversification of protein sources [5]. Beyond their nutritional value, proteins also provide key techno-functional properties in formulated foods, including emulsification, gelling, foaming, and water/oil binding [6].

BSG is a lignocellulosic biomass with a heterogeneous, rigid matrix that includes proteins, lignin, and structural polysaccharides. Although values vary with barley variety and brewing conditions, typical dry-basis compositions reported for BSG are ~18–25% protein, ~15–30% cellulose, ~19–20% hemicellulose, and ~11–23% lignin [3,5]. Importantly, beyond its protein fraction, BSG is also a concentrated source of cell-wall polysaccharides (often accounting for ~30–50% as dietary fibre), which can be valorised as functional fibres and as precursors of oligosaccharides [2].

In the last five years, different valorisation routes for BSG have been explored, with most studies focusing on protein extraction [5,7,8,9] and on the recovery of polysaccharides such as arabinoxylans [10,11] and β-glucans [12]. Conventional BSG protein recovery is commonly performed by alkaline solubilization, followed by protein precipitation (e.g., by isoelectric adjustment) and solid–liquid separation [13,14,15,16]. While alkaline extraction is straightforward and can yield meaningful protein recoveries, the recalcitrant lignocellulosic matrix can limit protein mass transfer, thereby slowing extraction kinetics and potentially constraining yield at practical processing times [4,5].

To address these limitations, recent research has explored emerging strategies to intensify BSG protein extraction while preserving or tailoring functionality. Physical intensification approaches, such as high-intensity ultrasound, pulsed electric fields (PEF), microwave-assisted extraction and ohmic heating, have shown potential to increase protein recovery under milder pH/temperature regimes compared with conventional controls [4,8,17]. Several studies confirmed that power ultrasound can markedly intensify BSG protein extraction under alkaline conditions. Li et al. [16] reported an optimised ultrasound-assisted extraction using 110 mM NaOH at 25 °C, applying probe ultrasound (250 W, 20 min, 60% duty cycle), achieving a protein extraction yield (before precipitation) of 86% (with 58% purity after downstream steps). In a more recent work, Ribeiro-Sanches et al. [18] applied ultrasound during alkaline extraction at pH 12 (NaOH), using 1200 W (pulsed 1 s on/1 s off) for 30 min while keeping the system at ~25 °C, and obtained a substantially higher protein recovery (72%, purity of 47%) than the conventional control (31%, purity of 54%). Finally, Mikkelsen et al. [8] showed that even milder media can benefit from ultrasound: using water extraction at pH 9 (≤50 °C), ultrasound achieved 59% protein recovery (purity of 25%).

Many studies assessed BSG protein extraction performance using empirical/statistical optimisation approaches (e.g., design of experiments and regression models such as RSM (response surface methodology) or systematic factor screening), as they describe factor effects and support prediction and optimisation with fewer experiments [15,16,17,19,20]. However, time-resolved kinetic studies of protein release from BSG are comparatively scarce [21,22,23]. In ultrasound-assisted extraction, time is typically included only as an optimisation factor (e.g., Li et al. [16] evaluated sonication time under alkaline ultrasound-assisted extraction), rather than reporting kinetic profiles. To date, a dedicated kinetic study for power-ultrasound extraction of BSG proteins has not yet been reported; such kinetic information could help identify rate-limiting regimes, justify time selection, and improve scale-up and process design.

In parallel to protein-focused extraction studies, integrated biorefinery approaches have been proposed to maximise BSG valorisation by recovering multiple fractions and generating co-products beyond a single protein isolate [5,24,25]. Representative examples include wet fractionation routes that intentionally split BSG into protein and fibre-enriched streams [26] and strategies linking protein recovery with changes in lignin-associated fractions [27]. Along the same lines, Belardi et al. [5] proposed a wet-BSG sequential concept combining enzymatic protein solubilization under mild conditions with the recovery of additional soluble fractions (e.g., phenolics) and a subsequent hydrothermal step to obtain oligosaccharides, thereby moving toward multi-product valorisation.

Despite these advances, studies that explicitly evaluate and/or revalorise the post-extraction solid residue after protein extraction remain limited. The above works [26,27,28] provide valuable examples of integrating protein extraction with information on other fractions, yet in most research, residue characterisation is often not reported or is only addressed superficially [8,17,18]. This point is especially relevant for ultrasound-assisted extraction. To the best of our knowledge, the extent to which sonication modifies the compositional profile of the remaining solid (e.g., lignin content and structural carbohydrate distribution) has not yet been systematically investigated.

Overall, the present work aims to explore how ultrasound affects BSG protein extraction and the transformation of residue composition. For this, we evaluated ultrasound-assisted extraction using mechanical agitation as a control, characterised the extracts (yield, emulsifying properties, and secondary structure), and assessed the composition of the post-extraction residue. A preliminary kinetic study at 25 and 50 °C was carried out to support the selection of extraction time and temperature. The novelty of this work lies in integrating mathematical modelling with product functional characterisation and residue assessment, thereby providing a broader perspective on ultrasound and developing an efficient, functional dual valorisation process.

## 2. Materials and Methods

### 2.1. Reagents

Distilled water (conductivity < 5 µS/cm) was used throughout the study. Sulfuric acid (96–98% *w*/*w*) was purchased from Scharlau (Barcelona, Spain). For monosaccharide determination, the standards arabinose, xylose, glucose, galactose, mannose, and rhamnose, as well as 2-deoxyglucose (≥98%), used as the internal standard, were obtained from Merck (Barcelona, Spain). Ammonia solution (32% *v*/*v*, analytical grade), sodium borohydride (≥98%), and 1-methylimidazole (≥99%) were also supplied by Merck (Barcelona, Spain). Acetic anhydride and glacial acetic acid were purchased from PanReac AppliChem (Barcelona, Spain), whereas dichloromethane (GC analysis grade) was obtained from Scharlau (Barcelona, Spain). Sodium tetraborate decahydrate (99.5% *w*/*w*) and 3-phenylphenol (85% *w*/*w*) were purchased from Sigma-Aldrich (Madrid, Spain). Glucuronic acid (≥98%) used for the uronic acid calibration curve was obtained from Merck (Barcelona, Spain). Sodium hydroxide (pellets) and hydrochloric acid (37% *w*/*w*, extra pure) were purchased from Scharlau (Barcelona, Spain). Bovine serum albumin (BSA, ≥98.5%), used as the standard for protein quantification by the Bradford assay, together with Coomassie Brilliant Blue G-250, orthophosphoric acid (85% *w*/*w*), and ethanol (96% *v*/*v*), were obtained from Merck (Barcelona, Spain). Sunflower oil from a local supermarket was used in this study (HACENDADO, Mercadona, Valencia, Spain).

### 2.2. Raw Matter

Brewer’s spent grain (BSG) was supplied by La Somniada Cooperativa V.—Cervecería L’Audaç (Valencia, Spain). The BSG originated from the production of a speciality beer brewed using Pilsen, Caragold, Biscuit and Munich malts, with a small proportion of chestnut. Fresh BSG was collected immediately after mashing and stored at −18 °C until use. Then, BSG was dried in a vacuum oven (60 °C, 24 h, 18–24 kPa absolute pressure), milled using an analytical mill (Yellowline by IKA, model A10, Staufen, Germany) and subsequently sieved through a 400 µm mesh. The mill was operated with small loads (ca. 30–50 g per batch) to avoid overloading and minimise heat build-up. No external cooling was applied, and no noticeable overheating of the material was observed during processing. Material loss during milling and sieving was negligible. After processing, all batches were combined in a single container and manually homogenised with a spatula. The resulting powder was vacuum-packed in a transparent, food-grade PA/PE (polyamide/polyethene) vacuum bag, protected from light, and stored at −18 °C until protein extraction experiments, then sieved (<400 µm). Particle size distribution of the BSG was determined in duplicate by dry sieving using a mechanical sieve shaker (FIT-0200, Filtra, Barcelona, Spain). A set of sieves with decreasing aperture size (0.500, 0.355, 0.180, and 0.045 mm) was used. The sieve stack was shaken for 5 min at power level 9 and cycle 9. After sieving, the mass retained on each sieve and in the bottom pan was weighed, and the results were expressed as retained mass percentage for each size fraction. The particle size percentiles D10, D50, and D90 were estimated from the cumulative passing distribution by interpolation, yielding values of 0.067 ± 0.004, 0.222 ± 0.003, and 0.3848 ± 0.0006 mm, respectively. The retained fraction and cumulative passing distribution are presented in Supplementary File S1.

#### Raw Matter Characterisation

Moisture content was determined gravimetrically according to the AOAC method 935.29 [29]. Briefly, 4–5 g of BSG were dried at 103 °C to constant mass, and results were expressed on a dry matter (dm). The proximate composition of the raw BSG (protein, fat, total carbohydrates, dietary fibre, and starch) was determined by an external accredited laboratory (Eurofins Ecosur S.A.U., Murcia, Spain) under ISO/IEC 17025 [30]. Total nitrogen was quantified by the Dumas combustion method, and protein content was calculated using a conversion factor of 6.25. Total fat was determined using a microwave-assisted extraction method. Dietary fibre was measured by an enzymatic–gravimetric procedure, and starch content was determined by an enzymatic method. Soluble sugars were analysed by IC-PAD (ion chromatography with pulsed amperometric detection) (internal method CR-TM5724). In addition to the outsourced analysis, the residue was further characterised in terms of lignin and structural carbohydrates (neutral sugars and uronic acids).

Total lignin was quantified as the sum of acid-insoluble lignin (InsLignin) and acid-soluble lignin (SolLignin) after two-step sulfuric acid hydrolysis following the method described by Sluiter et al. [31]. Briefly, samples (300 ± 10 mg, in triplicate) (the exact weight was recorded and used for calculation) were placed in 50 mL borosilicate glass autoclave bottles (GL 45 thread), loosely capped with their corresponding screw caps and treated with 72% (*w*/*w*) H_2_SO_4_ (3 mL). Suspensions were mixed for 1 min and incubated at 30 °C for 1 h with controlled magnetic agitation (80 rpm). The acid was diluted to 4% (*w*/*w*) with distilled water, then autoclaved at 121 °C for 1 h. After cooling, hydrolysates were vacuum-filtered through glass-fibre filters (1.6 μm pore size). The solid residues were washed with distilled water and dried at 105 °C for a minimum of 4 h to constant mass (less than ± 0.3 mg change in the weight) to determine InsLignin gravimetrically using an analytical balance (KERN, ABS 220-4N, Balingen, Germany).

For SolLignin, the hydrolysis liquor (before washing) was collected, and its absorbance was measured at 205 nm (UV–2401PC, Shimadzu, Tokyo, Japan) at 1 cm path length. Liquors were diluted with 4% *w*/*w* H_2_SO_4_ to obtain absorbance values below 1.0. SolLignin was calculated using the Beer–Lambert law with an extinction coefficient of 110 L g^−1^ cm^−1^ [32]. The UV-based determination of SolLignin should be considered an estimate, since other compounds may also absorb at 205 nm, and the use of a single absorptivity coefficient is an approximation [33].

Neutral sugars: arabinose (Ara), xylose (Xyl), glucose (Glc), galactose (Gal), mannose (Man), rhamnose (Rha), and fucose (Fuc) were quantified by GC–FID (Gas chromatography with flame ionisation detector) as alditol acetates following the procedure described by Gonzalez-Centeno et al. [34]. Briefly, after hydrolysis, 200 μL of 2-deoxyglucose (1 mg mL^−1^) was added to 1.1 mL of each sample as an internal standard for the quantitative determination. Quantification was based on individual calibration curves for each monosaccharide, relating the analyte-to-internal-standard peak area ratio to the corresponding mass ratio (Supplementary File S2).

Subsequently, 620 μL of hydrolysate was neutralised with 50 μL of 25% NH_3_. Then, 100 μL of 3 M NH_3_ containing 15% (*w*/*v*) NaBH_4_ was added as a reducing agent to convert the sugars into alditols by reducing the aldehyde groups to alcohols. The mixture was incubated at 30 °C for 1 h. After cooling, 450 μL of 1-methylimidazole was added as a catalyst, followed by 3 mL of acetic anhydride. The mixture was homogenised and incubated at 30 °C for 30 min to obtain the corresponding alditol acetates, which were subsequently extracted with dichloromethane. The organic phase containing the alditol acetates was separated by centrifugation, and the aqueous phase was removed by aspiration. Dichloromethane was then evaporated under a stream of argon at 40 °C.

The derivatised neutral sugars, converted into their corresponding alditol acetates, were separated isothermally at 220 °C by gas chromatography using a Hewlett-Packard 5890A system (Hewlett-Packard, Waldbronn, Germany) equipped with an OV-225 column packed with 3% Chromosorb WHP 100/120. Argon was used as the carrier gas at a flow rate of 20 mL min^−1^. Injector and FID detector temperatures were set at 230 and 240 °C, respectively.

Uronic acids were quantified colourimetrically [35]. 5 ± 1 mg (the exact weight was recorded and used for calculation) of the sample were treated with 72% (*w*/*w*) H_2_SO_4_ (200 µL) and incubated for 3 h at room temperature (22–25 °C). The hydrolysate was diluted with distilled water to a final acid concentration of 0.3% *w*/*w* and heated at 100 °C for 1 h in Pyrex tubes with screw caps. Samples were diluted to a final volume of 6 mL. Aliquots (0.5 mL) were treated with 3 mL of Na_2_B_4_O_7_·10H_2_O-sulfuric acid solution, heated at 100 °C for 10 min in Pyrex tubes with no screw cap, cooled in an ice bath, and then a 3-phenylphenol solution (0.15% *w*/*v* in 0.5 M NaOH; 100 µL) was added. After 1 h in the dark (22–25 °C), absorbance was read at 520 nm against a reagent blank (UV–2401PC, Shimadzu, Tokyo, Japan). Uronic acids were quantified by interpolation from a glucuronic acid calibration curve (Supplementary File S2).

Since arabinoxylans are the most abundant polysaccharides of the hemicellulose fraction in BSG [10,36,37], they were estimated (g/100 g dm) from the anhydro-corrected amounts of xylose and arabinose [31] according to Equation (1).

(1) Arabinoxylans=0.88·(Xyl)+0.88·(Ara)

where each monosaccharide is expressed as a mass percentage on a dm.

### 2.3. Protein Extraction

Two extraction techniques were tested (mechanical agitation, AG, and power ultrasound, US). Proteins were extracted from BSG in an alkaline solution. BSG powder was dispersed in 110 mM NaOH at solid-to-liquid ratios of 1:15 (8 g of BSG in 120 mL of NaOH solution) [16].

#### 2.3.1. Protein Extraction Under Mechanical Agitation

Extractions under mechanical agitation were carried out in a 250 mL beaker immersed in a thermostatic bath to maintain the selected temperature. The suspensions were mechanically stirred with an L-shaped stirring head (RZR 1, Heidolph, Schwabach, Germany) at 1200 rpm.

#### 2.3.2. Ultrasound-Assisted Extraction

A probe-type ultrasound device (UP400St, Hielscher, Germany), which operates at a nominal frequency of 24 kHz with automatic frequency tuning, was fitted with a 22 mm sonotrode and operated at 100% amplitude and 60% duty cycle (nominal power 260 W). To prevent bulk heating, sonication was performed in a jacketed glass vessel connected to a recirculating chiller, which continuously circulated coolant (ethylene glycol) through the vessel jacket to control temperature.

The real acoustic power delivered to the extraction medium was determined by calorimetry. The temperature of the extraction solvent (110 mM NaOH; no solids; without temperature control) was recorded every second for the first 5 min of sonication using a data logger (N2014, Comark, Norwich, UK) under the same ultrasound settings used for extraction. Acoustic power (P, W) was calculated from Equation (2).

(2) $$P=m\cdot Cp\frac{dT}{dt}$$

where *m* is the mass of solvent (kg), *Cp* is its specific heat capacity (J kg^−1^ °C^−1^), and *dT/dt* is the initial temperature slope (°C s^−1^). Acoustic power density (W L^−1^) was obtained by dividing P by the solvent volume. Under the conditions used, the acoustic power density was 500 ± 5 W L^−1^.

#### 2.3.3. Preliminary Kinetic Study of Protein

A preliminary kinetic study was carried out as an initial step to compare the extraction behaviour under ultrasound and mechanical agitation and to support the selection of time and temperature for the subsequent experiments. Protein extraction kinetics were evaluated in alkaline medium as a preliminary study to quantify the effect of the extraction temperature and the application of power ultrasound. Two extraction temperatures (25 and 50 °C) were tested. All kinetic experiments were carried out at least in triplicate. During extraction, 1 mL aliquots of the liquid phase were withdrawn at 3, 6, 10, 15, 20, 25, 30 and 40 min. Each aliquot was immediately diluted (1:6, *v*/*v*; 1 mL sample + 5 mL distilled water), manually mixed, and filtered through a 0.45 µm nylon syringe filter. Filtered samples were stored at −18 °C and analysed for protein content by the Bradford method within 3 days of collection.

The Bradford assay was used to quantify proteins in kinetic aliquots because it provides a rapid, sensitive determination of soluble protein in small sample volumes. Bovine serum albumin (BSA) was used as a standard. A stock solution (1 mg mL^−1^) was prepared by dissolving 10 mg BSA in distilled water and making up to 10 mL. Calibration standards were prepared over the range 0–1000 µg mL^−1^. Bradford reagent was prepared by dissolving 100 mg of Coomassie Brilliant Blue G-250 in 50 mL of 96% ethanol and 100 mL of 85% phosphoric acid. The solution was then transferred to a 1 L volumetric flask protected from light, and the volume was made up to 1 L with distilled water. For both standards and samples, 0.1 mL of solution was mixed with 5 mL of Bradford reagent, which had been prepared and filtered (1.6 μm pore size), and the mixture was vortexed. After colour development, 200 µL were transferred to a non-sterile 96-well polypropylene microplate with round U-bottom wells (up to 300 µL per well). Then, absorbance was measured at 595 nm using a microplate reader (EZ Read 2000, Biochrom, Cambridge, UK). Standards and samples were analysed in triplicate.

For the preliminary kinetic study, the Weibull model (Equation (3)) was used to describe the mass-transfer behaviour during protein extraction from BSG under both mechanical agitation and ultrasound-assisted conditions. This model has been widely applied to simulate solid–liquid extraction from plant materials [38,39].

(3) $$Y\left(t\right)=Y_{eq}(1-e^{{\left[-t/\alpha \right]}^{\beta }})$$

where Y(t) is the extraction yield at time t, $Y_{eq}$ is the equilibrium extraction yield, and α and β are model parameters. In this framework, α is a characteristic time parameter, with lower values indicating faster extraction kinetics. In contrast, β is a shape parameter related to the extraction curve profile. The temperature dependence of α  was described with an Arrhenius-type relationship (Equation (4)):

(4) $$\alpha =\alpha_{0}\;e^{\frac{Ea}{RT}}$$

where $\alpha_{0}$ is the pre-exponential factor, $E_{a}$ is the activation energy (J·${mol}^{-1}$), R is the universal gas constant (8.314 J·${mol}^{-1}$·$K^{-1}$), and T is the absolute temperature (K) [39].

When a temperature effect on extraction yields was observed, *Y_eq_* was assumed to vary linearly with temperature, as previously reported in other studies [38,39].

Parameter estimation was performed in three steps. First, Equation (3) was fitted separately to each extraction curve obtained at a fixed temperature using nonlinear least-squares regression in MATLAB R2026a Curve Fitter. This provided preliminary estimates of $Y_{eq}$, α, and β for each condition. For unconstrained fits, the Levenberg–Marquardt algorithm was used; for fits with parameter bounds, a trust-region–reflective algorithm was used.

Second, when a temperature effect was observed, the dependence of α and/or $Y_{eq}$ on temperature was analysed using linear regressions (ln(α) vs. 1/T and/or $Y_{eq}$ vs. T), to obtain initial estimates of $\alpha_{0}$, $E_{a}$, $Y_{eq0}$, and c. Finally, all experimental data obtained at different extraction times and temperatures were simultaneously fitted using a custom temperature-dependent Weibull equation in MATLAB Curve Fitter, including all adjustable parameters. These initial estimates were used as starting values and to define appropriate coefficient constraints to facilitate model convergence.

#### 2.3.4. Protein Extraction Experiments at the Selected Conditions

Based on the preliminary kinetic study (Section 2.3.3), the extraction conditions selected as a practical compromise between protein recovery and processing time were 20 min, and 25 °C. The rationale for this selection is discussed in the Results section, based on the preliminary kinetic results (Section “Mathematical Modelling”). Under these conditions, a single extraction cycle was compared with two consecutive cycles using fresh solvent, and both mechanical agitation (AG) and power ultrasound (US) were evaluated. The experimental set-up, solid-to-liquid ratio, and solvent composition were the same as those used in the kinetic study.

For these experiments, after extraction, suspensions were centrifuged (8000× *g*, 20 min, 10 °C) and the supernatant was recovered by decantation. When two extraction cycles were applied, the solid residue was resuspended in fresh 110 mM NaOH (same volume of solvent, 120 mL) and extracted again under the same conditions; both supernatants were pooled.

Proteins were precipitated by cooling the pooled supernatants in an ice bath (~10 °C) and adjusting pH to 3.8 by dropwise addition of HCl (1–4 M) under stirring [16]. The precipitated proteins were recovered by centrifugation (8000× *g*, 20 min, 10 °C). The pellet was resuspended in distilled water (80 mL), homogenised (30 s, 13,500 rpm) with an Ultra-Turrax (DI 25 basic, IKA, Staufen im Breisgau, Germany), and neutralised to pH 7.0 with 1 M NaOH. The neutralised protein dispersion was frozen and freeze-dried (4 days) using a LyoMicron Coolvacuum (Barcelona, Spain) at −80 °C and 0.15 mbar. to obtain protein-rich concentrates. This freeze-dried material is hereafter referred to as a protein-rich extract and coded as EX–N, where E denotes extract, X indicates the extraction technique (AG for agitation or US for ultrasound), and N is the number of extraction cycles (1 or 2).

On the other hand, the solid residues obtained after protein extraction were resuspended in distilled water to remove residual NaOH and then centrifuged (8000× *g*, 20 min, 10 °C). The recovered pellet was collected and retained for subsequent compositional analyses. These samples were hereafter labelled as RX–N, where R denotes residue, X indicates the extraction technique (AG for agitation or US for ultrasound), and N is the number of extraction cycles (1 or 2).

### 2.4. Extraction Yields and Characterisation of the Protein-Rich Extracts

The moisture content of the protein-rich extracts was determined as previously described in Section 2.2 [29]. The dry weight of the protein-rich extract was calculated considering its moisture content. From that value, the mass yield of the extraction was obtained using Equation (5).

(5) $$Mass\;Yield\;\left(\%\right)=\frac{m_{extract}\;}{m_{BSG}}\cdot \;100$$

where m_extract_ (g) is the dry mass of the freeze-dried protein-rich extract and m_BSG_ (g) is the dry mass of the raw BSG initially employed in the extraction.

The total protein content of the protein-rich extracts, expressed in g/100 g dm, was determined as previously described in Section 2.2 for the BSG. Finally, the protein yield was calculated as follows:

(6) $$Protein\;Yield\;(\%)=Mass\;Yield\frac{{Protein\;content}_{extract}}{{Protein\;content}_{BSG}}\cdot \;100$$

#### 2.4.1. Emulsifying Activity

The emulsifying activity and the emulsion stability were determined using a turbidimetric method [40]. Protein dispersions were prepared by dissolving isolate powder (0.25 g) in distilled water (25 mL) and stirring for 1 h. An emulsion was formed by mixing 6 mL of protein dispersion with 2 mL of sunflower oil and homogenising with an Ultra-Turrax (DI 25 Basic, IKA, Staufen im Breisgau, Germany) (30 s, 13,500 rpm). Immediately after homogenisation, an aliquot (20 µL) was diluted in 1 g/L sodium dodecyl sulfate (SDS; 1.5 mL) to arrest flocculation. Turbidity was monitored at 500 nm immediately after the sample preparation and after 10 min.

The emulsifying activity index (EAI, m^2^·g^−1^) was calculated from the turbidity (T) according to:

(7) $$EAI\;\left(\frac{m^{2}}{g}\right)=\frac{2\cdot 2.303\cdot A_{0}\cdot F}{c\cdot L\cdot \varphi }$$

where A_0_ is the absorbance at 500 nm, F is the dilution factor (76), c is the protein-rich extract concentration in the aqueous phase (g·m^−3^), L is the optical path length (m), and φ is the oil volume fraction in the emulsion. The emulsion stability index (ESI, min) was calculated as:

(8) $$ESI\;(min)=\frac{A_{0}}{A_{0}-A_{10}}\Delta t$$

where A_0_ and A_10_ are the absorbances at 0 and 10 min, respectively, and Δt = 10 min.

#### 2.4.2. Chemical Fingerprinting and Secondary Structure from FTIR Spectra

Protein-rich extracts were analysed by ATR-FTIR using a Bruker IFS66 spectrometer (Bruker, Billerica, MA, USA) over the 500–4000 cm^−1^ wavenumber range with 16 scans. Fourier self-deconvolution and second-derivative analysis were applied to enhance band resolution [41] with OMNIC E.S.P. 5.1 software, and peaks within the amide I region (1700–1600 cm^−1^) were identified. These peaks were assigned to protein secondary-structure elements as described by Ribeiro et al. [18], who also analysed proteins coming from BSG: β-sheet (1600–1640 cm^−1^), random coil (1640–1650 cm^−1^), α-helix (1650–1660 cm^−1^), and β-turn (1660–1700 cm^−1^). The area of each assigned band was integrated, and the relative content of each secondary-structure element was expressed as a percentage of the total amide I area.

### 2.5. Characterisation of the Extraction Residues

A compositional analysis of the extraction residues was performed, including protein, lignin, neutral sugars, uronic acids, and hemicellulose contents, following the same analytical procedures described for raw BSG characterisation (Section 2.2).

### 2.6. Statistical Analysis

All results are reported as mean ± standard deviation. Statistically significant differences (*p* < 0.05) are denoted by different letters. Since normality tests within each group have low power at small sample sizes, model assumptions were evaluated using model residuals. Residual normality was assessed by Q–Q plots and the Shapiro–Wilk test, while homogeneity of variances was evaluated using Levene’s test with the Brown–Forsythe correction. Data were analysed by analysis of variance (ANOVA) followed by Tukey’s honestly significant difference (HSD) test. A two-way ANOVA was applied to assess the effects of extraction technique (mechanical agitation vs. ultrasound) and the number of extraction cycles (1 vs. 2). When only main effects were significant, Tukey’s HSD was used for pairwise comparisons among factor levels; when a significant interaction was detected, post hoc comparisons were performed on the simple effects within the interaction. Effect sizes were estimated using partial eta squared (ηp^2^). All analyses were performed in R (v4.5.0, April 2025) using RStudio (v2025.05.1, June 2025) with the *agricolae* and *multcomp* packages [42,43].

## 3. Results

### 3.1. Raw Matter Composition

The composition of the dried BSG used as the raw material for the extraction experiments is summarised in Table 1. Starch was the predominant component (~37 g/100 g dm), followed by dietary fibre (~25 g/100 g dm). Protein also accounted for a substantial fraction (~16 g/100 g dm), whereas lipids and soluble sugars were present at comparatively low levels. Overall, these values are broadly consistent with ranges reported for BSG in the literature [4,5,8], except for the comparatively high starch content: several studies report only 2–7 g/100 g dm [4,5]. Nevertheless, high residual starch has been described for BSG from local craft (small-scale) brewing operations; for example, Jin et al. [44] reported 19–47 g/100 g dm and linked this to lower brewhouse efficiency associated with factors such as coarse milling and limited sparging, which can leave more endosperm material in the spent grain. In our case, the inclusion of a small proportion of chestnut as an adjunct may also have contributed to residual starch. These observations highlight the need for case-by-case characterisation, since BSG is a heterogeneous by-product whose composition depends strongly on raw materials and brewing conditions.

Glucose was the most abundant monosaccharide (42 ± 2 g/100 g dm for BSG), followed by xylose, uronic acids, and arabinose. Because starch accounted for 37 ± 1 g/100 g dm and is entirely composed of glucose units, approximately 88% of the measured glucose can be attributed to starch. Consequently, the non-starch glucose fraction (and thus cellulose and possibly hemicellulose) was not an abundant polysaccharide. As expected for BSG, the hemicellulosic fraction was mainly represented by arabinoxylan-type sugars, with xylose and arabinose as the dominant neutral monosaccharides, whereas other hemicellulose-associated sugars such as mannose and galactose were present only in minor amounts [10,36,37]. The presence of UA could be associated with both hemicellulose and pectic polysaccharides [45]. Based on the xylose and arabinose content, the estimated arabinoxylan content was 9.9 ± 0.2 g/100 g dm, which is lower than the value reported by Ribeiro et al. [18] (~30 g/100 g dm), likely reflecting differences in feedstock. Total lignin was 16 ± 1 g/100 g dm, in line with commonly reported values for BSG (~17–20 g/100 g dm) [5,18,26].

### 3.2. Preliminary Kinetic Study of Protein Extraction

**Figure 1:**
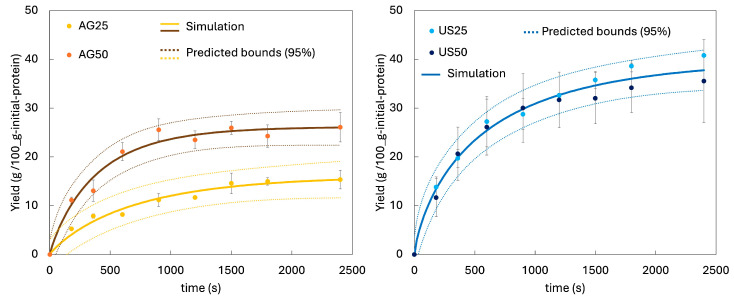
Experimental and calculated protein extraction curves from brewer’s spent grain (BSG) assisted by mechanical agitation (AG) and power ultrasound (US) at 25 and 50 °C. Dots correspond to experimental protein yields (mean ± SD). Solid lines represent the Weibull model fit, and dashed lines indicate the 95% prediction intervals.

The protein experimental extraction curves are shown in Figure 1 (dots) as mean ± SD. At 25 °C. In this preliminary kinetic study, the measured response corresponded to solubilised protein in the extraction liquor, as determined by the Bradford method. Ultrasound application clearly enhanced protein extraction compared with mechanical agitation. For example, after 40 min and 25 °C, the yield obtained with ultrasound (40 ± 1 g/100 g initial protein) was 2.5-fold higher than that achieved with agitation. This agrees with previous studies that compared extractions performed with and without ultrasound, consistently reporting improved protein recovery/yield when ultrasound was applied [8,16,18]. Overall, both our data and the literature support that ultrasound enhances mass transfer and matrix disruption, thereby increasing the amount of extractable protein [46]. However, at 50 °C, ultrasound still gave higher mean extraction yields than mechanical agitation, but the advantage was much smaller than that observed at 25 °C.

When it comes to temperature, it strongly affected the mechanically agitated extractions: operating at 50 °C produced clearly higher yields than at 25 °C (at 40 min, the yield at 50 °C was 1.7-fold higher than at 25 °C). Consistent with our results, Connolly et al. [13] also reported a strong temperature dependence in the alkaline extraction of BSG proteins under gentle stirring. Using 110 mM NaOH, raising the extraction temperature from 20 to 50 °C increased the recovery of the original protein in pale BSG from 29 ± 1% to 59 ± 5% (~2.1-fold). Moderate heating seems to enhance alkaline protein solubilization and mass transfer.

In contrast, temperature had no meaningful effect on the ultrasound-assisted process; increasing the temperature from 25 to 50 °C did not improve the yield and, overall, no significant differences (*p* > 0.05) were observed between the two extraction curves.

The limited response of ultrasound-assisted extraction to increasing temperature from 25 to 50 °C may be explained by the coexistence of different effects. First, although the bulk temperature was controlled (the vessel was kept under external cooling/thermostatic control), ultrasound can still heat the medium during sonication. In probe systems, local temperature gradients (“hot spots”) may form near the sonotrode, so the temperature close to the probe can transiently exceed the set point [47]. This is relevant because reviews on ultrasound processing of plant proteins report a measurable temperature rise during sonication and indicate that extraction temperatures above ~60 °C are generally undesirable due to their negative impact on protein structure [48]. Second, increasing temperature raises vapour pressure and can reduce cavitation intensity, thereby weakening the mechanical effects of ultrasound [49]. Consequently, at a nominal 50 °C, local overheating may promote denaturation/aggregation that offsets further solubilization, while weaker cavitation reduces the ultrasound advantage over agitation. Moreover, because ultrasound already strongly enhances mass transfer at 25 °C, further increasing temperature may provide little additional benefit once diffusion is no longer the main limiting step.

To our knowledge, studies specifically assessing the interaction between ultrasound and elevated temperature for BSG protein extraction are scarce. However, a similar trend has been reported for other matrices: in ultrasound-assisted alkaline extraction of defatted rice bran protein, varying temperature between 25 and 45 °C did not significantly (*p* > 0.05) affect extraction performance, suggesting that once ultrasound is applied, moderate temperature increases may not translate into higher yields [50].

#### Mathematical Modelling

The Weibull model was used to describe the protein extraction curves from BSG under mechanical agitation and ultrasound-assisted conditions. Using the experimental yields obtained at different extraction times, the Weibull parameters *α*, *β* and the equilibrium yield (Y_eq_) were preliminarily identified for each set of operating conditions.

For the AG curves, α was assumed to follow an Arrhenius-type temperature dependence (Equation (4)), whereas the equilibrium yield (Y_eq_) was assumed to vary linearly with temperature (Equation (9)):

(9) $$Y_{eq}=cT+Y_{eq0}$$

where *c* and *Y_eq_*_0_ are empirical constants. Consequently, the kinetics of agitation-assisted extraction were described by simultaneously identifying five parameters: *α*_0_ (pre-exponential factor), $E_{a}$ (activation energy), β, c and *Y_eq_*_0_.

In contrast, ultrasound-assisted extraction curves displayed a markedly different behaviour. Under these conditions, neither α nor $Y_{eq}$ exhibited a significant temperature dependence within the studied range. Therefore, ultrasound-assisted extraction kinetics were adequately characterised by estimating only three parameters: α, β and $Y_{eq}$.

These estimated parameters are shown in Table 2, together with the corresponding prediction intervals (95%).

As shown in Table 2, the proposed model showed an excellent goodness of fit, with high R^2^ values (AG: 0.980, US: 0.983) and similarly high adjusted R^2^ (AG: 0.974, US: 0.981), indicating that they explain most of the experimental variability without signs of overfitting. In addition, the low RMSE values (AG: 1.39, US: 1.68) suggest small prediction errors, supporting the adequacy of the proposed models to describe the extraction kinetics. This agreement is also evident in Figure 1, where the simulation (continuous lines) is compared with the experimental results (dots).

For mechanically agitated kinetics (AG), the Weibull parameter α, calculated from the Arrhenius-type relationship using the fitted $\alpha_{0}$ and $E_{a}$ values, decreased from 702 s at 25 °C to 389 s at 50 °C, indicating a faster approach to equilibrium at higher temperature. In contrast, in the ultrasound experiments α remained approximately constant (~598 s) within the studied conditions. At 25 °C, ultrasound reduced α compared with AG, confirming an acceleration of the extraction kinetics. Because α represents a characteristic time scale (smaller α implies a faster approach to $Y_{eq}$), these results suggest that temperature accelerated extraction under AG, whereas its effect became negligible when ultrasound was applied. Likewise, the kinetic enhancement provided by ultrasound at 25 °C was not observed at 50 °C, where α did not decrease. As previously discussed, this may be related to a reduced cavitation intensity at higher temperatures (higher vapour pressure leading to less energetic collapses), possible protein denaturation/aggregation and associated solubility changes [51], and/or mass-transfer limitations arising from an earlier approach to liquid-phase saturation under ultrasound-assisted extraction.

Regarding the apparent activation energy, $E_{a}$ could only be estimated for the mechanically agitated extractions (AG), since the ultrasound-assisted kinetics did not show a measurable dependence on temperature within the studied range. To the best of our knowledge, no studies have reported $E_{a}$ specifically for protein extraction kinetics from brewer’s spent grain (BSG). Nevertheless, our fitted value (18.89 kJ/mol) is comparable to values reported for protein recovery from other plant matrices. For example, Yang et al. [52] and Zhang et al. [53] studied protein extraction from peanut and soybean flours, respectively, using an AOT reverse-micelle system in which proteins are transferred from the solid phase into an organic phase containing surfactant micelles. Using an Arrhenius-type analysis, they reported apparent activation energies of 10.0 kJ/mol (peanut) and 3.4–6.0 kJ/mol (soybean), which are of the same order of magnitude but lower than the value obtained here for alkaline solid–liquid extraction under mechanical agitation. According to the classification proposed by Wang et al. [54], activation energies below 20 kJ/mol are typically associated with diffusion-controlled extraction, whereas values between 20 and 40 kJ/mol indicate a mixed diffusion–reaction regime. In this context, the estimated $E_{a}$ (18.9 kJ/mol) suggests that protein extraction under agitation was mainly governed by diffusion in the 25–50 °C range.

The Weibull shape parameter β provides insight into the curvature of the extraction profiles. In all cases, β was below 1, indicating a decelerating extraction behaviour, i.e., the extraction rate decreased with time. Consistently, the kinetic curves (Figure 1) became markedly concave after approximately 20 min. This behaviour is typical of solid–liquid extraction processes, where an initial fast “washing/desorption” step is progressively replaced by a slower, diffusion-controlled stage as readily accessible proteins are depleted and the driving force for mass transfer decreases [54].

Regarding the equilibrium yield, $Y_{eq}$ increased from 16 to 26 g/100 g initial protein for the mechanically agitated extractions (AG) when the temperature rose from 25 to 50 °C. In contrast, the ultrasound-assisted runs reached a higher equilibrium value, $Y_{eq}\;$40 ± 3 g/100 g initial protein, suggesting that ultrasound promoted the release of protein fractions that remained poorly accessible under mechanical agitation alone. Under all conditions, the plateau was reached within ~40 min.

A comparable trend has been reported for other protein systems. Li et al. [51] investigated ultrasound pretreatment of an aqueous rice-protein suspension to enhance protein solubilization into water and modelled the kinetics using a modified Weibull/first-order approach that accounts for aggregation under severe sonication. They observed that the equilibrium (plateau) soluble-protein yield increased with both ultrasound intensity and temperature; for example, after 18 min, it rose from 0.338% to 0.391% when ultrasonic power density increased from 45.5 to 51.8 W/L at 303 K, and from 0.338% to 0.386% when temperature increased from 303 to 313 K.

Overall, the kinetic modelling indicates that higher temperature accelerated extraction under AG, whereas ultrasound, compared with AG, increased both the extraction rate and $Y_{eq}$. Therefore, ultrasound not only accelerated the approach to equilibrium, but also increased the amount of extractable/solubilizable protein recovered. This result is consistent with the mechanisms commonly proposed for ultrasound-assisted extraction from plant matrices, whereby acoustic cavitation induces pore formation, surface erosion, and particle fragmentation, thus increasing surface area and solvent penetration, and enhancing mass transfer at the solid–liquid interface [55].

Based on the kinetic modelling, the second part of the study was designed to operate at 25 °C because it is a mild condition that avoids potential thermal effects on proteins and, as previously mentioned, ultrasound-assisted kinetics showed no noticeable acceleration with temperature within the explored range.

Moreover, the extraction time per cycle was set to 20 min. In all cases, the Weibull fits gave β < 1, indicating a decelerating extraction rate. Consistently, the experimental curves became clearly concave after ~20 min, so extending the extraction time yielded diminishing returns. To address this, we implemented solvent renewal to restore the concentration driving force and reduce liquid-phase saturation effects. Accordingly, we carried out one and two consecutive 20 min cycles, using fresh solvent in each cycle. This approach allowed us to quantify the additional protein recovered after the first cycle, once the readily accessible fraction had been removed. Parallel extractions under mechanical agitation were performed as controls to quantify the incremental effect of ultrasound.

### 3.3. Extracts Characterisation

#### 3.3.1. Extraction Yields

The statistical analysis results of the extraction yields and the protein-rich extracts characteristics are summarised in Table 3.

**Figure 2:**
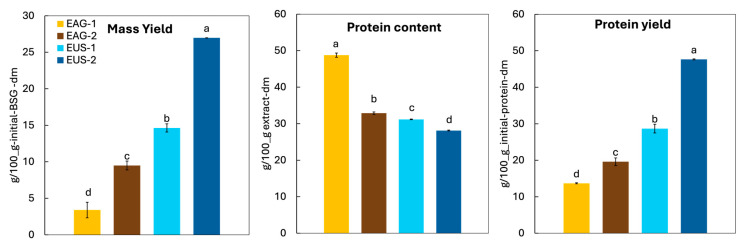
Mass yield, protein content of the extract, and protein yield obtained at 25 °C using mechanical agitation (AG) or ultrasound assistance (US), after one (1) or two (2) consecutive extraction cycles. Different letters for the same variable indicate significant differences among treatments (*p* < 0.05).

The extraction performance at 25 °C is summarised in Figure 2, which compares ultrasound assistance (US) with mechanical agitation (AG), as well as single-cycle and two consecutive extraction cycles using fresh solvent in each cycle. Results are reported using three complementary indicators to capture different aspects of the process: mass yield (overall solubilization/mass transfer from BSG to the liquid phase), protein content of the extract (selectivity and therefore “purity” of the recovered solids), and protein yield (overall protein recovery, calculated relative to the initial protein present in BSG).

Regarding mass yield, it was significantly affected by the extraction technique, the number of cycles, and their interaction (ηp^2^ > 0.89, *p* < 0.05, Table 3). In both one- and two-cycle extractions, ultrasound increased mass transfer compared with mechanical agitation. For example, the mass yield for EUS-1 (15.5 ± 0.6 g/100 g BSG) was 4.3-fold higher than EAG-1, indicating a stronger release/solubilization of BSG components under sonication. The highest value was obtained for EUS-2 (28.49 ± 0.02 g/100 g dm), which was 2.8-fold higher than EAG-2. The number of extraction cycles had a strong effect for both techniques: EAG-2 yielded 2.8-fold more than EAG-1, whereas the increase with ultrasound was smaller, with EUS-2 being about 1.8-fold higher than EUS-1.

Regarding extract purity (protein content), the extraction technique, the number of cycles, and their interaction all had significant effects (ηp^2^ > 0.99, *p* < 0.05, Table 3). The highest protein contents were obtained under mechanical agitation (AG). Specifically, EAG-1 extracts showed a 56% higher protein content than US-1 (31.18 ± 0.01 g/100 g extract). This difference narrowed in the two-cycle extractions: the protein content of EAG-2 decreased to 32.87 ± 0.02 g/100 g extract, remaining only 16% higher than EUS-2. Although ultrasound can enhance matrix disruption and improve protein recovery, it may not be selective for proteins, reducing extract purity by promoting the co-extraction of non-protein components (e.g., soluble carbohydrates) and increasing sample complexity, unless additional purification steps (e.g., dialysis) are applied.

The marked drop from EAG-1 to EAG-2 (~73%) indicates that, during the second cycle, compounds other than proteins were co-extracted from BSG, reducing extract purity. In contrast, ultrasound-assisted extraction showed a much smaller decrease between cycles (~11%), suggesting a more stable extract composition.

Finally, regarding protein yield, the two studied factors and their interaction had significant effects (ηp^2^ > 0.96, *p* < 0.05, Table 3). As expected, ultrasound-assisted extraction provided the highest yields, consistent with its higher mass recovery. The maximum yield was obtained for EUS-2 (47.6 ± 0.1 g/100 g dm), which was 2.4-fold higher than that of EAG-2; a similarly large difference was observed for the single-cycle extractions (2.1-fold, EUS-1 vs. AG-1). The number of cycles also had a marked impact: moving from one to two cycles increased the yield by 1.4-fold under agitation (EAG-2 vs. EAG-1) and by 1.7-fold under ultrasound (EUS-2 vs. EUS-1).

Reported protein extraction yields from BSG vary widely in the literature, ranging from about 11% under conventional alkaline extraction of black BSG [13] to 86% under ultrasound-assisted alkaline extraction [16], highlighting substantial differences in raw material, extraction conditions, and processing strategy. The yields obtained in the present work (14–20% for AG and 29–48% for US) are close to those of Mikkelsen et al. [8], who evaluated several emerging extraction technologies under relatively mild conditions (pH 6 or 9 and temperatures below 50 °C) and reported protein recoveries of 29.5–37.5% for the controls and 36.5–58.6% for ultrasound-assisted extraction.

Higher values have been reported in other studies, but these were generally achieved using longer and/or more complex protocols. For example, Belardi et al. [5] reported 65% protein extraction from wet BSG using alcalase at pH 8.5 and 40 °C, but the enzymatic hydrolysis lasted 4 h. Li et al. [16] also reported a high yield (86.2%) under ultrasound-assisted alkaline extraction in 110 mM NaOH at 25 °C for 20 min (2 cycles); however, direct comparison is not straightforward because their protein yield was expressed based on the protein present in the supernatant relative to the protein in the initial dry BSG, rather than on the protein finally recovered in the dried fraction. In comparison with other technologies, such as pulsed electric field (PEF), the extraction yields obtained in the present study were higher than those reported by Paksin et al. [17], who achieved a maximum protein recovery of 24% by optimizing pulse number (5000–9000), electric field strength (8–10 kV/cm), and frequency (8–10 Hz). Nevertheless, their extracts showed higher protein purities, reaching up to 89%. Comparing with high hydrostatic pressure, Gokulakrishnan et al. [56] reported protein extraction yields from BSG up to 44% at 600 MPa, which are comparable to those obtained here for US, although the resulting extracts again showed higher protein purity (56%).

Previous studies indicate that process intensification can increase extraction yield while decreasing the protein fraction due to co-extraction of non-protein components. For instance, Ribeiro-Sanches et al. [18] showed that adding alkaline hydrogen peroxide increased extraction yield from 31% (conventional extraction using 2 M NaOH) to 52%, but decreased the protein content of the recovered BSG protein concentrate from 54 to 46 g/100 g dm; moreover, when combining hydrogen peroxide and ultrasound application they achieved the highest yield (74%) while maintaining a lower protein content (47 g/100 g dm) than the conventional control, which the authors attributed to enhanced transfer of structural carbohydrates and lipids into the product (i.e., a dilution effect).

In line with this yield-purity trade-off, Mikkelsen et al. [8] reported that ultrasound at pH 9 produced high protein recovery (59%) but a relatively low protein content in the crude extract (24%). Overall, ultrasound enhances mass transfer and thereby increases extraction yield; however, because it is not selective for proteins, additional purification steps may be required, such as additional washes [57] or ultrafiltration [58]. Further studies addressing the economic feasibility and environmental impact of these processes are also needed to enable a more comprehensive comparison with other technologies.

#### 3.3.2. Emulsifying Activity

**Figure 3:**
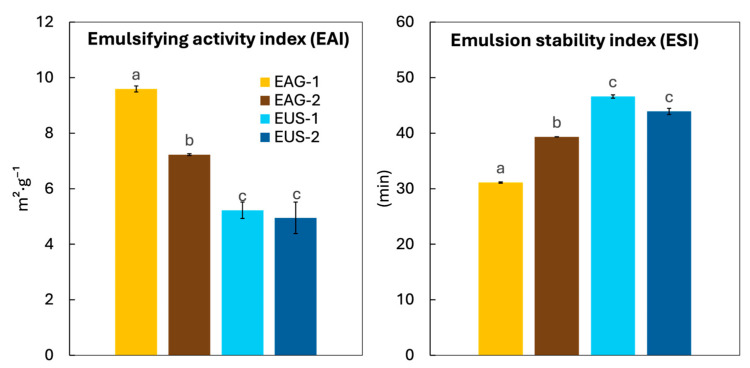
Emulsifying activity index (EAI, m^2^·g^−1^) and emulsion stability (ES, (10 min)) of BSG protein-rich extracts obtained at 25 °C using mechanical agitation (AG) or ultrasound assistance (US), after one (1) or two (2) consecutive extraction cycles. Different letters for the same variable indicate significant differences among treatments (*p* < 0.05).

Proteins are widely used as food emulsifiers due to their amphiphilic nature, which enables adsorption at the oil–water interface and promotes interfacial film formation [59]. Accordingly, the emulsifying activity index (EAI) was determined for the protein-rich extracts. The extraction technique (ηp^2^ = 0.975), the number of cycles (ηp^2^ = 0.862), and their interaction (ηp^2^ = 0.797) significantly (*p* < 0.05) influenced EAI, with the extraction technique the most influential parameter (Table 3). As shown in Figure 3, extracts obtained by ultrasound assistance exhibited significantly lower EAI than those obtained under mechanical agitation. The highest EAI was observed for EAG-1 (9.6 ± 0.1 m^2^·g^−1^), which was ~84% higher than the EAI of EUS-1, while this value for EAG-2 (7.23 ± 0.03 m^2^·g^−1^) was ~46% higher than for EUS-2. The number of cycles affected EAI mainly under agitation, with a ~25% decrease from EAG-1 to EAG-2, whereas EAI did not significantly (*p* > 0.05) change between EUS-1 and EUS-2.

Overall, this trend is consistent with differences in extract composition: the extracts with higher protein content exhibited higher EAI (AG extracts), suggesting that ultrasound promoted the co-extraction of non-protein compounds that dilute and/or interfere with the interfacial active protein fraction. Literature values for BSG-derived proteins typically fall within the range of 15–40 m^2^·g^−1^ [16,18,60]; however, most studies report EAI per gram of protein, whereas in the present study, EAI is expressed per gram of freeze-dried extract, which is more relevant for practical ingredient applications. When normalizing our results to the protein fraction, EAI increases to 17–22 m^2^·g^−1^ protein (AG 20–22; US 17–18 m^2^·g^−1^), which is comparable to the reported values and still indicates slightly lower interfacial activity in the ultrasound extracts, potentially due to partial denaturation and/or aggregation, as well as competitive adsorption effects.

In contrast, ESI (emulsion stability index) (Figure 3) was higher for ultrasound-derived extracts. For example, EUS-1 showed ~50% higher stability than EAG-1 (46 vs. 31 min). This suggests that co-extracted components, although they do not exhibit strong interfacial activity themselves, may contribute to stabilisation by increasing the viscosity of the continuous phase and/or providing steric hindrance, mechanisms commonly associated with polysaccharides and other soluble matrix compounds [61]. When comparing the values obtained for protein extracted in one or two cycles, the EAI was 26% higher for EAG-2 than for EAG-1. EAG-2 also showed less purity than EAG-1, and probably the presence of other compounds was able to maintain the emulsion stable. The number of cycles had no significant effect on the ESI of the ultrasound-derived extracts (*p* > 0.05).

#### 3.3.3. Chemical Fingerprinting and Secondary Structure from FTIR Spectra

**Figure 4:**
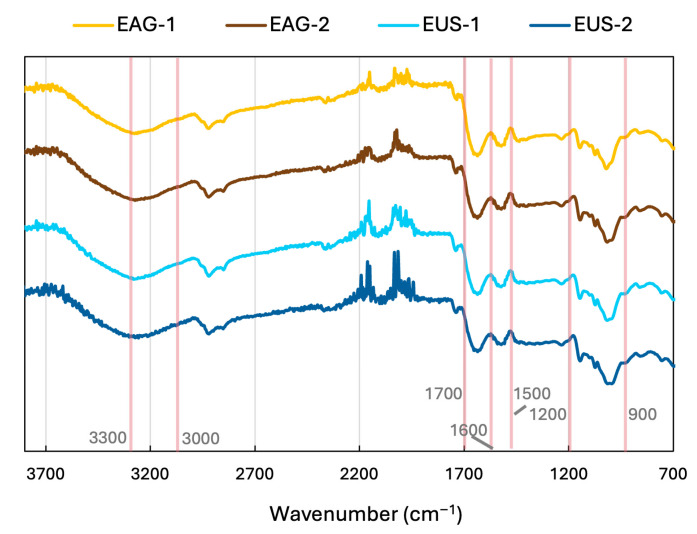
FTIR spectra of BSG protein-rich extracts obtained at 25 °C using mechanical agitation (AG) or ultrasound assistance (US), after one (1) or two (2) consecutive extraction cycles.

The FTIR spectra of the BSG protein extracts (Figure 4) showed the characteristic protein bands together with a signal attributable to co-extracted matrix components. A broad absorption around ~3300 cm^−1^ is assigned to overlapping O–H stretching (bound water and hydroxyl groups from carbohydrates) and N–H stretching (Amide A) from proteins [62,63], with the exact shape influenced by hydrogen bonding.

The region ~2920 and ~2850 cm^−1^ corresponds to C–H stretching of aliphatic –CH_2_/–CH_3_ groups, commonly associated with lipids and hydrophobic moieties in biopolymers [63]. In the mid-infrared protein region, the Amide I band (1700–1600 cm^−1^) (mainly C=O stretching of the peptide bond) and Amide II band (1550–1500 cm^−1^) (N–H bending coupled with C–N stretching) confirm the presence of protein as the dominant macromolecule in the concentrates [62].

Finally, the strong “fingerprint” region between ~1200 and 900 cm^−1^ is dominated by C–O and C–O–C vibrations typical of polysaccharides (e.g., residual hemicellulosic/starch structures) [64]. Overall, the spectra indicate a protein-rich material with detectable aliphatic (lipid/hydrophobic) and carbohydrate signatures.

**Figure 5:**
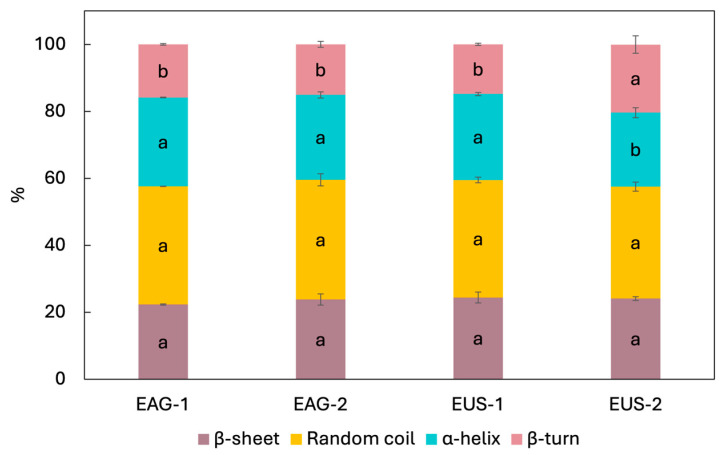
Secondary structure of the protein extracted using mechanical agitation (AG) or ultrasound assistance (US), after one (1) or two (2) consecutive extraction cycles. Different letters for the same variable indicate significant differences among treatments (*p* < 0.05).

The Amide I region (1700–1600 cm^−1^) was further analysed to assess protein secondary structure, and the results are presented in Figure 5. Overall, the extracts exhibited broadly similar secondary-structure profiles. Random coil was the predominant motif in all samples (33–36%), with no significant differences among treatments (*p* > 0.05). β-sheet contents were also comparable across extracts (22–24%), again with no significant differences (*p* > 0.05).

In contrast, the α-helix was significantly affected by the extraction technique, the number of cycles, and their interaction (*p* < 0.05, Table 3). Specifically, EUS-2 showed a slightly but significantly lower α-helix content (22 ± 1%) than the other samples (25.8 ± 0.6%). Consistently, EUS-2 exhibited a significantly higher β-turn proportion (20 ± 3%) compared with the remaining extracts (15.3 ± 0.5%).

The secondary-structure distribution observed here is in the range reported by Ribeiro et al. [18] for BSG proteins extracted using ultrasound and/or alkaline hydrogen peroxide (random coil 23–31%, β-sheet 23–37%, α-helix 13–19%, and β-turn 23–31%). These authors reported increases in β-turn and α-helix upon applying ultrasound and/or hydrogen peroxide, and attributed these changes to protein unfolding under strongly alkaline conditions and to ultrasound cavitation effects. In the present study, applying ultrasound for two cycles increased β-turn (in agreement with Ribeiro et al.) but decreased α-helix, highlighting that the direction of conformational changes may depend on treatment severity and extraction history. For instance, Rafique et al. [65] reported a non-monotonic response of α-helix in oat protein isolate across ultrasound intensities (200–600 W), supporting that ultrasound-induced restructuring is sensitive to processing intensity.

From a structural standpoint, α-helix and β-sheet are generally considered more ordered motifs, whereas β-turn and random coil represent less ordered conformations. Therefore, ultrasound treatment applied over two extraction cycles appears to promote a shift toward a more disordered and flexible protein conformation, likely through disruption and reorganisation of non-covalent interactions (e.g., hydrogen bonding and hydrophobic interactions) and altered aggregation/association equilibria [41].

### 3.4. Residue Characterisation

**Figure 6:**
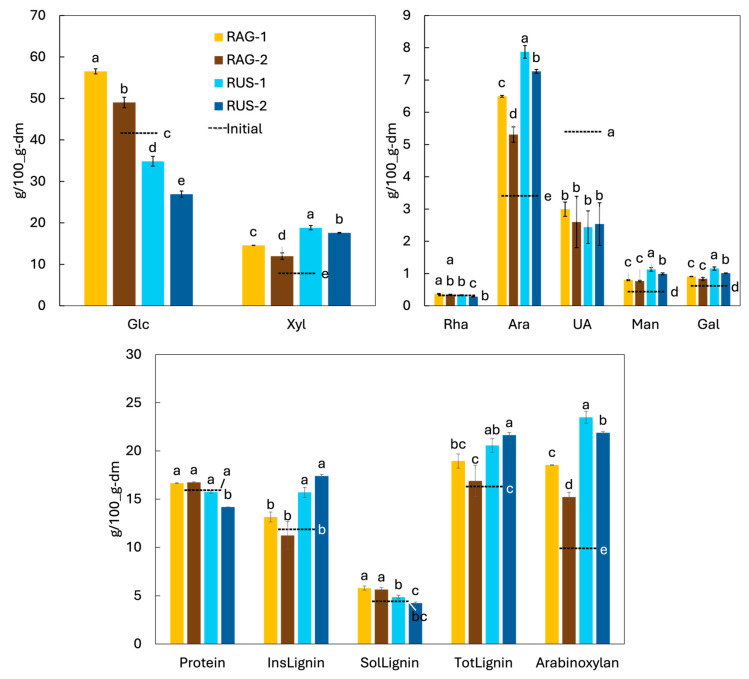
Composition of the BSG (initial) and the residues after the extraction of protein at 25 °C using mechanical agitation (AG) or ultrasound assistance (US), after one (1) or two (2) consecutive extraction cycles. Different letters for the same variable indicate significant differences among treatments (*p* < 0.05).

The monosaccharide composition of the solid residues after extraction is shown in Figure 6; the initial values for the BSG are included for reference. Glucose (Glc) remained the predominant monosaccharide in all samples. However, its proportion changed markedly depending on the extraction method. Residues obtained under mechanical agitation (AG) showed a significantly higher Glc content than the initial BSG, which is consistent with a relative enrichment of starch as other components were preferentially solubilised and removed. In contrast, ultrasound-derived residues (US) exhibited a clear decrease in Glc (compared with both the initial BSG and the AG residues), indicating that ultrasound promoted the transfer of starch-derived material into the alkaline liquid phase. In addition, Glc decreased from one to two extraction cycles for both techniques, with the lowest value observed for RUS-2 (26–27 g/100 g dm), corresponding to ~35% less Glc than in the initial BSG (42 ± 2 g/100 g dm). Since starch was the main contributor to Glc in the initial BSG, this decrease suggests that part of the starch fraction was co-extracted together with the proteins under ultrasound-assisted conditions. This behaviour may be related to the combined action of the alkaline medium, which can favour starch swelling and partial solubilisation, and the mechanical effects of acoustic cavitation, which may disrupt the residual matrix and increase the release of non-protein components. Therefore, these results support the idea that ultrasound enhanced protein recovery but at the expense of lower extract selectivity, as also reflected in the reduced protein purity of the ultrasound-derived extracts. In fact, ultrasound has been successfully applied to enhance starch recovery from several raw materials, including mango kernels [66] and corn [67,68]. In these studies, ultrasound-assisted processes increased starch extraction yield relative to conventional approaches such as wet milling, with reported improvements of approximately 7–28% [66,68]. The higher starch content in the ultrasound-derived extracts may help explain their higher emulsion stability index despite a lower emulsifying activity, since polysaccharides, although they typically show limited interfacial activity, can increase the viscosity of the continuous phase and provide steric hindrance, thereby reducing droplet migration and coalescence [69].

In parallel, xylose (Xyl) and arabinose (Ara) increased in all residues compared with the initial material. These monosaccharides are characteristic of cereal hemicelluloses, particularly arabinoxylans, which consist of a β-(1 → 4)-linked xylan (xylose) backbone substituted with arabinose residues [70]. Mannose (Man) and galactose (Gal) followed the same trend, although they were presented in much lower levels. Overall, arabinoxylans were more abundant in US residues than in AG residues (Figure 6). This can be interpreted as a “purification” of the structural polysaccharide fraction in the residue when ultrasound enhances the removal of starch. Accordingly, the highest arabinoxylans content was obtained for RUS-1 (23.5 ± 0.6 g/100 g dm), which was ~2.4-fold higher than in the initial BSG. Similar to our findings, Reis et al. [71] showed that an ultrasound pretreatment of BSG in water (750 W, 3–12 min) promoted starch removal, thereby enriching the hemicellulosic fraction, specifically arabinoxylans, in the remaining solid. He et al. [26] extracted protein from BSG using different chemicals (sodium hydroxide and sodium bisulfite) and enzymatic treatments (alcalase), and also observed that the crude fibre fraction increased in the extraction residue from around 23 to 75–85 g/100 g.

Nevertheless, arabinoxylans decreased in the residues from one to two cycles for both AG and US experiments, suggesting that the second extraction cycle may begin to solubilise this material. This is consistent with the use of aqueous alkali (e.g., NaOH/KOH) for the extraction of hemicellulosic fractions, since alkaline media swell the cell wall and cleave alkali-labile linkages (especially ester bonds) that connect hemicellulose to lignin and other wall components, thereby increasing hemicellulose solubility [72,73]. Stronger conditions and/or longer residence times can further promote dissolution but may also lead to chain scission and a reduction in molecular weight [74]. On the other hand, uronic acids (UA) were significantly lower in the residues than in the initial BSG and did not differ significantly among residues (*p* > 0.05), indicating that UA-containing components were preferentially removed during extraction.

The protein content of the residues was generally comparable to that of the initial BSG, which can be explained by the concurrent solubilization of non-protein components: although protein was extracted (particularly under US), the overall residue mass also decreased, leading to similar protein proportions in most cases. Only US-2 showed a significantly lower protein content than the initial BSG.

Finally, insoluble lignin (the dominant lignin fraction in BSG) showed a clear enrichment in the solid residues when applying ultrasounds, whereas the proportion of this compound did not significantly change for AG residues. Compared with the initial material (11.9 ± 0.8 g/100 g dm), insoluble lignin was about 39% higher in the ultrasound residues. This enrichment was expected because lignin is a highly recalcitrant aromatic polymer that forms a rigid cell-wall network, limiting matrix disassembly and making it comparatively difficult to remove under mild extraction conditions [75]. Therefore, the higher insoluble lignin values in the US residues likely reflected the extensive removal of other constituents (e.g., protein and starch), which increased the relative contribution of lignin in the remaining solids. In contrast, the acid-soluble lignin fraction was lower in the US residues than in the AG residues and remained close to (or slightly below) the initial value, suggesting that ultrasound may have favoured the transfer of acid-soluble lignin–type components to the alkaline liquor and/or altered this fraction during extraction. Total lignin followed the same pattern as insoluble lignin, with the highest values observed for the ultrasound-treated residues (RUS-1 and RUS-2; average 21.1 ± 0.7 g/100 g dm).

Overall, ultrasound generated a solid residue enriched in arabinoxylans. This arabinoxylans-rich material could be further valorised as a functional dietary fibre ingredient, for example, for water-binding and viscosity or texture modulation. These residues could be specifically upgraded through controlled modification strategies. In particular, partial hydrolysis could be applied to produce xylo- and arabinoxylo-oligosaccharides, which have shown prebiotic potential [70]. Likewise, targeted enzymatic or mild physicochemical treatments could be used to increase arabinoxylan accessibility, improve solubility, and tailor molecular weight, thereby enhancing their functionality as fibre ingredients [76]. From a technological and sustainability perspective, arabinoxylans have also been explored for applications as adhesives, thickeners, stabilisers, and emulsifiers [77], and more recently as film-forming materials [10].

## 4. Conclusions

Power ultrasound proved to be an effective intensification strategy for alkaline protein extraction from brewer’s spent grain under mild conditions. At 25 °C, ultrasound increased the 40 min protein yield to ~40 g/100 g initial protein compared with ~16 g/100 g initial protein under mechanical agitation. Based on the kinetic profiles, which reached a clear concave behaviour after ~20 min under ultrasound, short extraction times were selected to maximise productivity. Under the selected short extraction conditions (two consecutive 20 min cycles with solvent renewal), the highest recovery was obtained with ultrasound over two cycles (~48 g/100 g initial protein), which was ~2.4-fold higher than that achieved by agitation under the same scheme. Overall, these results indicate that high yields can be achieved at low temperatures, and that solvent renewal is an efficient strategy to recover an additional, less-accessible protein fraction without extending extraction time.

From a functional standpoint, extraction conditions also shaped extract performance: agitation produced higher purity and emulsifying activity, whereas ultrasound extracts showed higher emulsion stability, likely influenced by co-extracted macromolecules.

Beyond protein recovery, ultrasound supported a dual valorisation concept by modifying the composition of the residual solid. Ultrasound-treated residues were enriched in structural carbohydrates, particularly hemicellulose, largely due to the removal of starch-derived glucose. The hemicellulose content after one extraction cycle with ultrasound was ~2.4-fold higher than in the initial BSG. However, a second cycle reduced hemicellulose content under both agitation and ultrasound, suggesting that repeated alkaline exposure may begin to promote hemicellulose solubilization.

Overall, ultrasound-assisted alkaline extraction provides a short, low-temperature route to obtain (i) a protein-rich ingredient and (ii) a hemicellulose-enriched solid stream that can be further valorised as a functional fibre or as a feedstock for xylo-/arabino-xylo-oligosaccharides.

Future work should focus on improving extraction selectivity and validating industrial feasibility, including simple downstream purification strategies such as membrane filtration or diafiltration to increase protein purity, as well as process-level evaluation of energy demand, techno-economic performance, and functionality in representative food systems, and protein bioavailability.

## Figures and Tables

**Table 1:** Composition of the dried BSG.

Component *	(g/100 g dm)		
Water	0.7	±	0.2
Starch	37	±	1
Total dietary fibre	25.0	±	0.8
Protein	15.9	±	0.7
Fat	4.3	±	0.1
Ash	3.7	±	0.3
Soluble sugars	2.6	±	0.2
**Monosaccharides and uronic acids**			
Glc	42	±	2
Xyl	7.8	±	0.1
UA	5.4	±	0.3
Ara	3.4	±	0.1
Gal	0.62	±	0.02
Man	0.44	±	0.02
Rha	0.32	±	0.02
**Structural polysaccharides and lignin**			
Arabinoxylan	9.9	±	0.2
Insoluble lignin	11.9	±	0.9
Soluble lignin	4.4	±	0.2
Total lignin	16	±	1

* The three shown divisions are not additive.

**Table 2:** Estimated parameters of the modified Weibull model for protein extraction from BSG under mechanical agitation (AG) and of the standard Weibull model under ultrasound assistance (US), standard error of each parameter (SE) and the RMSE, R^2^, and adjusted R^2^ of the models.

	Parameter	Value	SE
**AG**	*α*_0_ (s)	0.3432	1.423
	$E_{a}$ (kJ/mol)	18.89	11
	β	0.9035	0.1585
	*c* (°C^−1^)	0.4023	0.0791
	*Y_eq_*_0_ (g/100 g initial protein)	6.055	3.8642
	RMSE = 1.39; R^2^ = 0.980; adjusted R^2^ = 0.974		
**US**	*α* (s)	598	124.7
	β	0.7474	0.1044
	*Y_eq_* (g/100 g initial protein)	40.14	2.9558
	RMSE = 1.68; R^2^ = 0.983; adjusted R^2^ = 0.981		

**Table 3:** Statistical F-values, *p* significance codes and η^2^p (effect size) from two-way ANOVA for the variables evaluated in the protein extraction process carried out with two different techniques (AG and US) with 1 or 2 consecutive 20 min extraction cycles.

	Technique			Cycles			Technique: Cycles		
Variable	F	*p*	ηp^2^	F	*p*	ηp^2^	F	*p*	ηp^2^
Mass Yield	1367.7	***	0.994	563.0	***	0.986	64.6	***	0.890
Protein Content	3307.2	***	0.998	2378.4	***	0.997	1091.8	***	0.993
Protein Yield	2163.9	***	0.996	723.1	***	0.989	199.1	***	0.961
Emulsion activity index	317.6	***	0.975	50.0	***	0.862	31.5	***	0.797
Emulsion stability index	15.1	**	0.653	1.1	ns	0.125	4.4	ns	0.357
Secondary structure									
β-sheet	2.8	ns	0.259	0.7	ns	0.078	1.7	ns	0.173
Random coil	3.2	ns	0.286	0.7	ns	0.082	2.3	ns	0.223
α-helix	15.4	**	0.658	21.0	**	0.724	5.3	*	0.400
β-turn	6.7	*	0.455	8.7	*	0.520	15.2	**	0.655

Significance codes: ***: *p* < 0.001; **: *p* < 0.01; *: *p* < 0.05; ns: not significant.

## Data Availability

The original contributions presented in this study are included in the article and Supplementary Materials. Further inquiries can be directed to the corresponding author.

## Supplementary Materials

The following supporting information can be downloaded at: https://www.mdpi.com/article/10.3390/foods15101701/s1, File S1: Particle size distribution of BSG determined by dry sieving. (a) retained mass percentage in each particle size range and (b) cumulative passing distribution. Error bars represent the standard deviation. File S2: Linear ranges, calibration equations, limits of detection (LOD), limits of quantification (LOQ), and coefficients of determination (R^2^) for the analytical methods used in this study.

## Abbreviations

The following abbreviations are used in this manuscript:

AG	mechanical agitation
ANOVA	analysis of variance
AOAC	Association of Official Analytical Chemists
ATR-FTIR	attenuated total reflectance Fourier-transform infrared spectroscopy
BSA	bovine serum albumin
BSG	brewer’s spent grain
D10	10th percentile of the particle size distribution
D50	50th percentile of the particle size distribution
D90	90th percentile of the particle size distribution
dm	dry matter (dry basis)
EAG-1	Extract obtained by mechanical agitation after one cycle
EAG-2	Extract obtained by mechanical agitation after two cycles
EAI	emulsion activity index
ESI	emulsion stability index
EUS-1	Extract obtained by ultrasound application (500 W L^−1^) after one cycle
EUS-2	Extract obtained by ultrasound application (500 W L^−1^) after two cycles
FID	flame ionisation detector
GC–FID	gas chromatography with flame ionisation detection
InsLignin	acid-insoluble lignin
PA/PE	polyamide/polyethene
PEF	pulsed electric fields
pH	negative logarithm of hydrogen ion activity
RAG-1	Residue obtained from the extraction with mechanical agitation after one cycle
RAG-2	Residue obtained from the extraction with mechanical agitation after two cycles
RMSE	root mean square error
RUS-1	Residue obtained from the extraction by ultrasound application after one cycle
RUS-2	Residue obtained from the extraction by ultrasound application after two cycles
RSM	response surface methodology
RZR 1	overhead mechanical stirrer model (Heidolph)
SD	standard deviation
SDS	sodium dodecyl sulfate
SE	standard error
SolLignin	acid-soluble lignin
UA	uronic acids
US	ultrasound
US-1	extract obtained by ultrasound after 1 extraction cycle
US-2	extract obtained by ultrasound after 2 extraction cycles
USA	United States of America
UV	ultraviolet (spectrophotometry)
UV–2401PC	UV–Vis spectrophotometer model (Shimadzu)
*v*/*v*	volume/volume
*w*/*v*	weight/volume
*w*/*w*	weight/weight
η^2^p	partial eta squared (effect size)
