# Supplementary material for: Brewer’s Spent Grain as a Source of Proteins and Valuable Polysaccharides
Source: Foods. 2026 May 12;15(10):1701. doi: 10.3390/foods15101701 (PMC13206433; doi:10.3390/foods15101701)
Supplement: Supplementary file 1 [file foods-15-01701-s001.zip › foods-4250194-supplementary/Supplementary File S1.pdf]

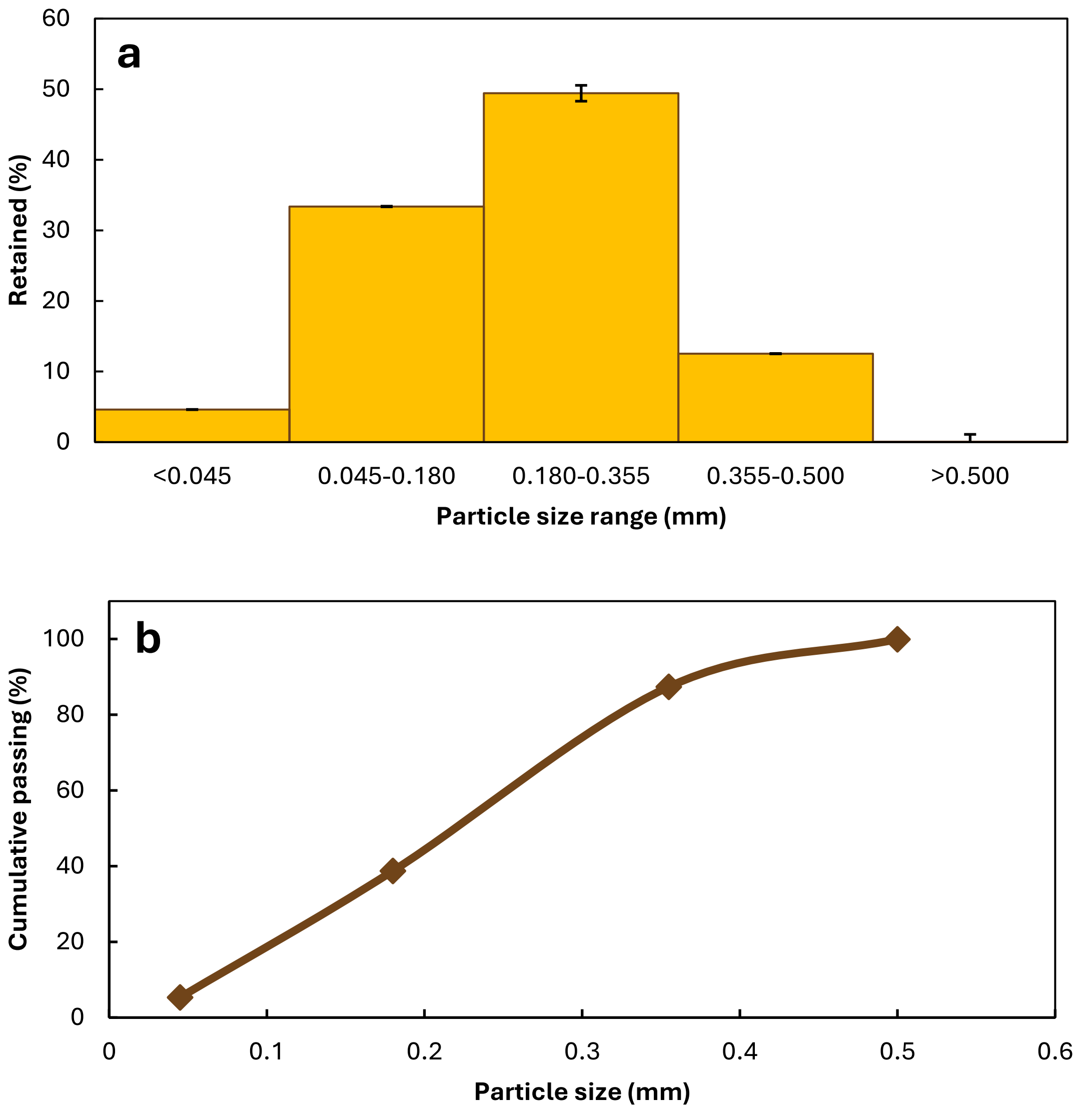

Supplementary 1. Particle size distribution of BSG determined by dry sieving. a) retained mass percentage in each particle size range and b) cumulative passing distribution. Error bars represent the standard deviation.
