# Supplementary material for: Brewer’s Spent Grain as a Source of Proteins and Valuable Polysaccharides
Source: Foods. 2026 May 12;15(10):1701. doi: 10.3390/foods15101701 (PMC13206433; doi:10.3390/foods15101701)
Supplement: Supplementary file 1 [file foods-15-01701-s001.zip › foods-4250194-supplementary/Supplementary File S2.pdf]

**Supplementary File S2**

Linear ranges, calibration equations, limits of detection (LOD), limits of quantification (LOQ), and coefficients of determination ($R^2$) for the analytical methods used in this study.

**Gas Chromatography method**

| | Slope $((A_{ns}/A_{std})/(m_{ns}/m_{std}))$ | LOD (mg/mL) | LOQ (mg/mL) | $R^2$ |
|---|---|---|---|---|
| Rha | 0.98 | 0.50 | 1.68 | 0.989 |
| Fuc | 0.97 | 0.19 | 0.65 | 0.998 |
| Ara | 1.08 | 0.29 | 0.97 | 0.996 |
| Man | 1.05 | 0.28 | 0.95 | 0.996 |
| Gal | 0.91 | 0.27 | 0.90 | 0.997 |
| Glc | 0.91 | 0.27 | 1.68 | 0.997 |

Linear range 0-400 mg/mL

**UV-Vis analytical methods**

| Lineality range | Compound | Slope (ppm/abs) | LOD (ppm) | LOQ (ppm) | $R^2$ |
|---|---|---|---|---|---|
| 0-80 ppm | Uronic acid | 0.00637 | 1,45 | 4.83 | 1.00 |
| 0-1000 ppm | Proteins (Bradford method) | $4.68x10^{-4}$ | 73.98 | 246.61 | 0.992 |

**Nomenclature**

| | |
|---|---|
| $A_{ns}$ | Peak area of the neutral sugar |
| $A_{std}$ | Peak area of the internal standard |
| Ara | Arabinose |
| Fuc | Fucose |
| Gal | Galactose |
| Glc | Glucose |
| LOD | Limit of detection |
| LOQ | Limit of quantification |
| Man | Manose |
| $m_{ns}$ | Mass of the neutral sugar |
| $m_{std}$ | Mass of the internal standard |

In all cases, the y-intercept was very close to zero and its 95% confidence interval included zero. Thus, the intercept was considered negligible for quantification purposes.
